# Treg cell therapy manufacturability: current state of the art, challenges and new opportunities

**DOI:** 10.3389/fimmu.2025.1604483

**Published:** 2025-05-23

**Authors:** Janelle Stoops, Tara Morton, James Powell, Amanda L. Pace, Jeffrey A. Bluestone

**Affiliations:** Process and Analytical Development, Sonoma Biotherapeutics, South San Francisco, CA, United States

**Keywords:** Treg - regulatory T cell, CAR (chimeric antigen receptor) T cell therapy, cell therapy manufacturing, TCR - T cell receptor, autoimmune diseases

## Abstract

Autologous cell therapy is a revolutionary new paradigm in medicine. Significant advancements in personalized therapeutic treatments with engineered T cells have been seen across the immuno-oncology markets. The global market is expanding as new cell types treat other conditions, like autoimmunity and transplant rejection. Key to the success of these novel cell therapies is manufacturability; ensuring robust processes that can reliably deliver treatments that meet the medical needs. Using the expertise and experience of the current state of Regulatory T cell (Treg) manufacturing at Sonoma Biotherapeutics as a prototypical case, we review manufacturing challenges and opportunities to ensure success.

## 1 Current state for Treg manufacturing

Regulatory T cells (Treg) is a novel cell therapy approach that is designed to control autoreactive, adaptive, and innate immune cells to rebalance autoimmunity, resulting in long term immune tolerance and healing. This product platform is built on decades of research including multiple founders of Sonoma Biotherapeutics, who are among the pioneers in Treg biology and cell therapy (1). If demonstrated to be clinically successful, Treg cell therapies are potentially applicable to multiple diseases: ranging from autoimmunity (e.g. Rheumatoid Arthritis, Multiple Sclerosis, Inflammatory Bowel Disease, Type 1 Diabetes, etc.), organ transplant rejection, neurogenerative, cardiovascular and virally induced inflammatory diseases. The core premise of Treg drug product success is that effective *ex vivo* expansion combined with genetic engineering can enhance Tregs efficacy in five complementary areas.

The Five Pillars and consequences for manufacturing:

**Selection and expansion**

The manufacturing process starts with the right cell and source of Treg material. Most efforts require the isolation of a rare but biologically validated isolation process to purify Tregs which are derived from the thymus and responsible for immune tolerance. Other approaches utilize Tregs isolated from leukapheresis material. Once isolated and engineered, the resulting antigen-specific Treg population must be expanded to a specific quantity to induce an efficacious immune system reset once reintroduced into the patient (2).

**Specificity**

The Treg needs to be properly engineered to hone to a specific disease-related target to shut down local inflammation in the absence of system suppression. To achieve this, once isolated, the purified cells can be engineered to express a specific antigen receptor, either T Cell Receptor (TCR) or Chimeric Antigen Receptor (CAR). This enhances the therapeutics specificity while significantly ameliorating the risk of contaminating T effector cells (Teff). This entails several genetic engineering steps that either utilize viral vectors or gene editing and require, in some cases, novel manufacturing approaches.

**Potency**

The goal of cell therapies is to develop products that can effectuate immune-based disease remission and tolerance induction following a single dose. To achieve this, the Tregs must maintain their identity, function, and stability to maintain their immunosuppressive function. The purpose of potency is to ensure the intended biological function of the Tregs are maintained. Thus, the manufacturing process must result in Treg products that retain critical quality attributes to maintain biological activity or potency. In this regard, it is important to note that the poly pharmaceutical nature of Treg activity impacts the ability to develop reliable potency assays as determination of which Treg activity. For instance, cytokine deprivation, suppressive cytokine production or modification of tissue metabolism is critical for any given disease setting. Thus, when considering potency, it will be necessary to consider multi-parameter analyses.

**Stability**

An engineered Treg must continue to express the genetic modification(s), that are responsible for enhanced specificity and/or functional activities (3). The risk of genomic (be it transcriptional or epigenetic) instability is a critical concern when developing a Treg cell product, as this can lead to conversion into Teff that might trigger an adverse pro-inflammatory response resulting in the destruction of the targeted tissues. In this regard, additional safety measures should be considered such as tagging features and suicide genes that will permit isolation of the product after dosing, phenotypic and functional monitoring and, if needed, elimination of the therapy (4).

**Persistence**

It is likely that the persistence of Treg cells *in vivo* is likely to depend on the engineered Treg trafficking to the right location and maintaining their functional activity for extended periods of time. Although there is ample pre-clinical and early clinical evidence that the therapeutic Treg cells can persist, the length required is yet untested and likely different in individual disease settings. In fact, the ability of Tregs to recruit resident cells into the regulatory network, so-call infectious tolerance (5, 6) may result in shorter-term persistence than anticipated. Of course, retreatment may also be possible should COGS and additional product production be feasibility.

The cell therapy development community across both academic and industry are actively advancing Treg cell therapy processes and manufacturing to advance the field. This review will highlight the opportunities to develop complex and tailored manufacturing approaches to ensure that reliable and reproducible products are generated (5).

### 1.1 Challenges in manufacturing (scalability, cost, dose enabling)

While the cell therapy industry has made great strides in establishing best practices and procedures resulting in the commercial success for autologous CAR-T cell therapy products, the T cell therapy manufacturing process is still in early stages with continued challenges (7). The engineered Treg manufacturing space is no exception. In fact, given the low percentage of Tregs in circulation and the lack of a single cell surface molecule that enables the selection of purified cells, the manufacturing process has proven to be among the most challenging of cell types. Isolating a specific cellular phenotype drastically limits the number of cells entering the manufacturing process. The cells must then be forward processed, genetically modified, and expanded to high yields to enable a therapeutic dose. Across the autologous cell therapy landscape there are three core universal challenges of living drug products to be addressed: Scalability, Dose determination and Cost.

At the forefront of engineered Treg manufacturing is the challenge of scalability. Current Treg manufacturing is labor-intensive and contains open manipulations with highly specialized equipment. Automation platforms, integrated unit operations and closed cell processing will be key as the industry moves towards improved efficiency and higher patient throughput (8). There are multiple efforts to create fully closed and automated systems for CAR-T manufacturing. However, in the engineered Treg manufacturing space, the requirement is not just enrichment but cell sorting for a pure population that is required. Sorting technology at a manufacturing scale is still new and creating a fully closed end-to-end system is premature. Thus, efforts are underway to automate or close individual unit operations within the manufacturing process and will still require work for seamless integration.

Dose enabling cell number is a critical aspect to consider when designing a cell therapy manufacturing platform for engineered Tregs. Autologous cell therapy utilizes cells from each individual patient (9) resulting in a patient specific drug product. The manufacturing process must be capable of withstanding process performance variability from an uncontrolled starting material (autologous blood product from each patient) and produce a high cell number for the final drug product. Treg cells do not expand at the same level as CD8+ CAR-T cells *in vivo*, so achieving dose levels for Tregs may be more critical. Cost is another key component of this novel pillar of medicine. Cell therapies are inherently expensive given the degree of specialized equipment, which includes single use raw materials, highly skilled manual labor and analytical testing to enable drug product lot disposition. Unlocking opportunities for cost savings will improve therapy accessibility and streamline development efforts (10).

## 2 Treg cell therapy manufacturing: state of the art

### 2.1 Overview of Treg cell therapy manufacturing

Autologous Treg Cell Therapy is a type of immunotherapy that involves isolating and expanding Treg cells from a patient’s own immune system to treat various autoimmune diseases, graft-versus-host disease (GVHD), or other conditions involving immune dysregulation. The efficiency and specificity of Treg based therapies depend heavily on the methods used for their isolation, activation, expansion and in some approaches the conversion into functional Tregs. Below we provide a high-level overview of methodologies employed across the field to optimize Treg therapies.

#### 2.1.1 Enriched populations with subsequent activation and expansion in the presence of rapamycin

Enrichment of Treg populations before activation is a critical step in ensuring that the expanded population retains its regulatory function. Typically, Tregs are enriched from peripheral blood mononuclear cells (PBMCs) or apheresis products using cell surface markers such as CD4, CD25, and/or CD127. This process is further refined by using rapamycin during expansion. Rapamycin is a well-established mTOR inhibitor that helps prevent the expansion of conventional effector T cells, which can otherwise confound the therapeutic effect of the Treg infusion (11).

Rapamycin works by selectively inhibiting the proliferation of Teffs while allowing for the robust expansion of Tregs. This selective expansion is important because Teffs, if expanded inadvertently, could counteract the immunosuppressive effects of the Tregs. In fact, some protocols specifically employ rapamycin in the culture medium during expansion to maintain the Treg phenotype and prevent the conversion of Tregs into potentially harmful effector cells. This strategy ensures that the final Treg product is both functional and of sufficient quantity for therapeutic application.

#### 2.1.2 Bead-based enrichment

Bead-based enrichment techniques offer a high-throughput and effective method for isolating Tregs from PBMCs. Magnetic beads conjugated with antibodies targeting Treg surface markers, such as CD25, can be used to isolate Tregs with a high degree of purity. This technique, when combined with sorting, can significantly increase the yield of Tregs, allowing for more efficient expansion and subsequent therapeutic use.

One advantage of bead-based enrichment is its ability to selectively isolate Tregs with minimal contamination from other cell types. Moreover, these beads can also be used in tandem with cytokine-based protocols or rapamycin to further promote the expansion and activation of Tregs. However, bead-based enrichment methods may sometimes result in the loss of certain subpopulations of Tregs, so optimization is required to ensure that the final Treg product maintains functional diversity (12).

#### 2.1.3 Flow-based cell separation techniques

Flow cytometry-based separation techniques, such as those using Tyto, BD or Sony sorters, are considered gold standards for isolating highly pure populations of Tregs. These techniques leverage specific surface markers (e.g., CD25, CD127, CD4) to isolate and sort Tregs with high precision.

Flow cytometry-based sorting can be more labor intensive and time consuming compared to bead-based methods, but it offers greater flexibility and precision in isolating Tregs with specific markers (7). It is also possible to isolate different subsets of Tregs based on surface markers, providing even more customization for therapeutic needs.

#### 2.1.4 Genetic engineering and in vitro transformation

Another innovative approach in Treg therapy involves transforming conventional T cells (CD4+ T cells) into Tregs using genetic engineering or culture with Treg promoting cytokines. One strategy is the genetic modification of T cells to express FOXP3. The introduction of FOXP3, often using viral vectors or CRISPR/Cas9 systems, reprograms conventional CD4+ T cells into induced Tregs (13).

While FOXP3 overexpression is highly effective at converting conventional T cells into a Treg like phenotype, the stability and long-term functionality of FOXP3 expressing cells can be a concern. For this reason, the use of cytokine driven *in vitro* culture methods has gained traction as an alternative or complementary approach. These cultures often include TGF-β which plays a critical role in driving the differentiation of Tregs from naïve CD4+ T cells. TGF-β induces FOXP3 expression, as well as other key Treg characteristics such as immune suppressive cytokine production and suppression of effector T cell function (13).

While there are various techniques to enrich, activate, and expand Tregs for therapy, the integration of these methods can be synergistic in developing effective and safe Treg based therapies. The remainder of this review will focus on one approach, Sonoma Biotherapeutics Treg manufacturing process to highlight our state-of-the-art manufacturing process experience and complexity of Treg cell therapy.

### 2.2 Sonoma Biotherapeutics Treg manufacturing platform: experience and opportunities

The autologous cell therapy manufacturing process developed by Sonoma Biotherapeutics begins with the collection of patient-derived material through leukapheresis. Patient information is verified, leukapheresis material is washed, and cells are prepared for enrichment. The Treg cells are first enriched with an initial cell separation technology which utilizes magnetic beads coated with antibodies specific to CD25 (interleukin 2α receptor chain), which is highly expressed on Treg cells. Following CD25 bead enrichment, a highly robust flow cytometry cell sorting technique using a cocktail of fluorochrome-coupled antibodies enables the separation of Tregs from other cell populations. The resulting process generally results in purities of greater than 95%. Once purified, the Tregs are activated using a bead-based expansion system, using CTS Dynabeads Treg Expander beads coated with anti-CD3/CD28. After 2–3 days, the Tregs are transduced with a lentiviral vector expressing chimeric antigen receptor (CAR) or T Cell Receptor (TCR) of choice, often with a tag or suicide gene, followed by expansion in a chemically defined cell culture medium plus IL-2 cytokine.

**Figure 1:**
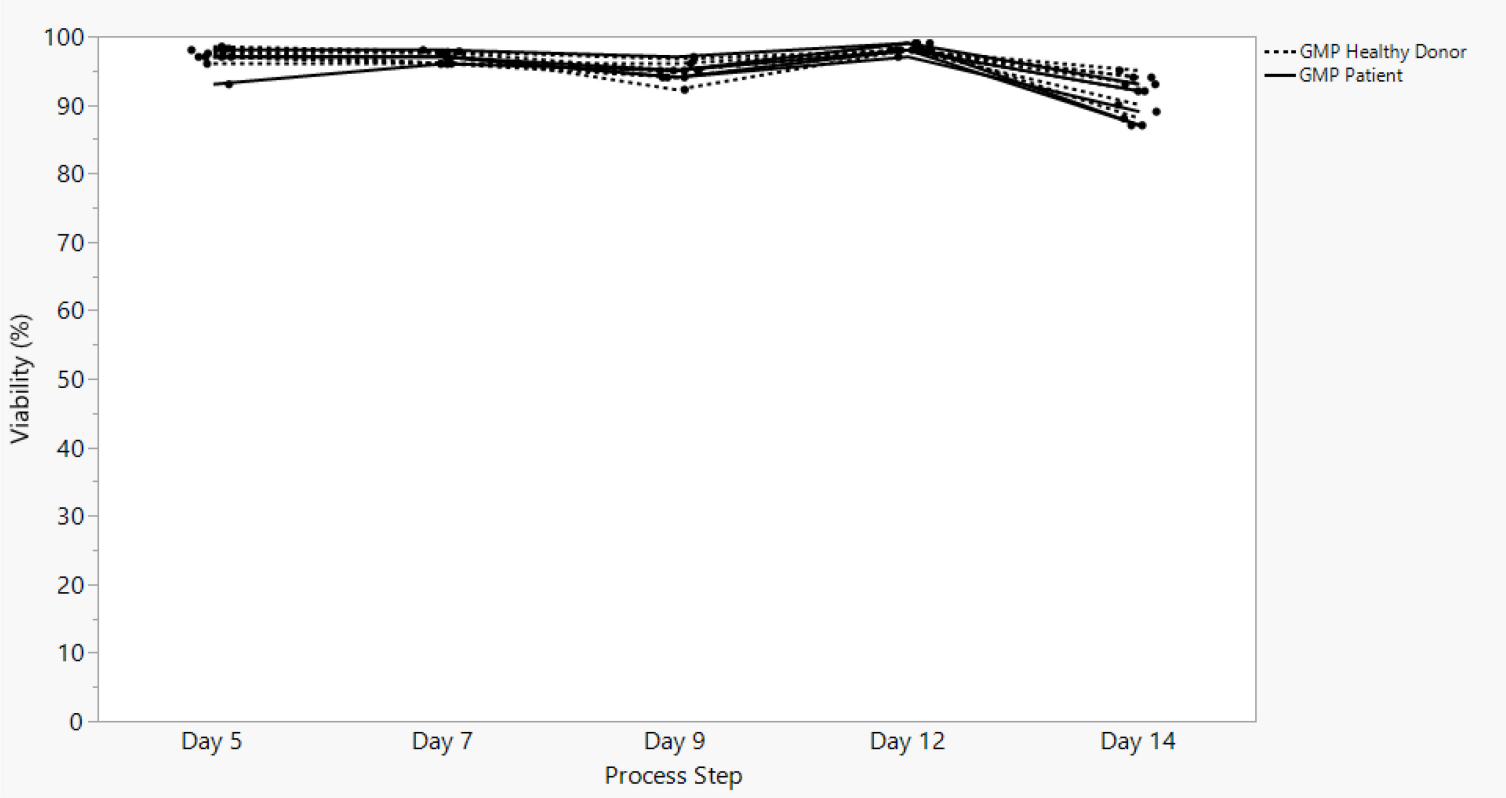
SBT-77–7101 manufacturing experience demonstrates highly viable products maintained throughout cell culture expansion.

**Figure 2:**
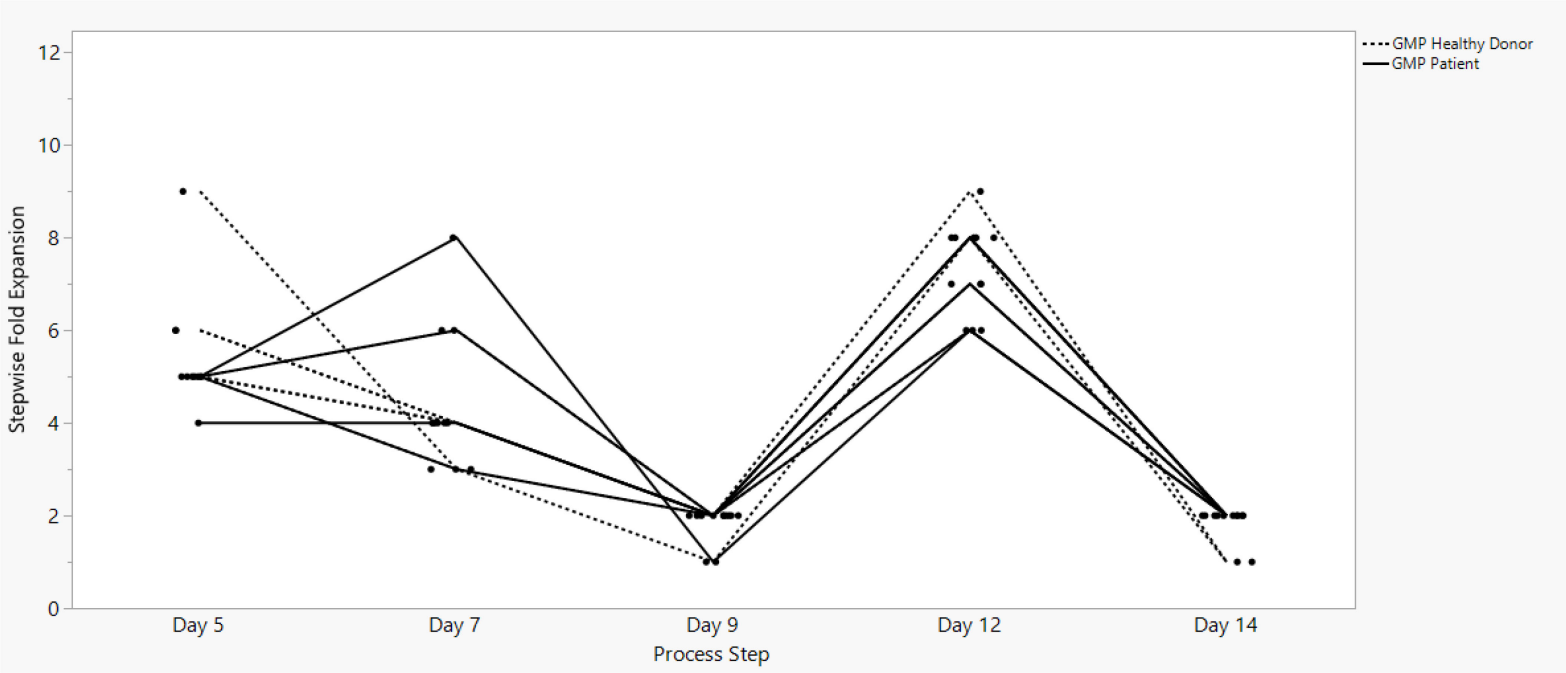
SBT-77–7101 manufacturing experience demonstrates continuous cell growth with incremental stepwise fold expansion throughout the entire expansion unit operation.

**Figure 3:**
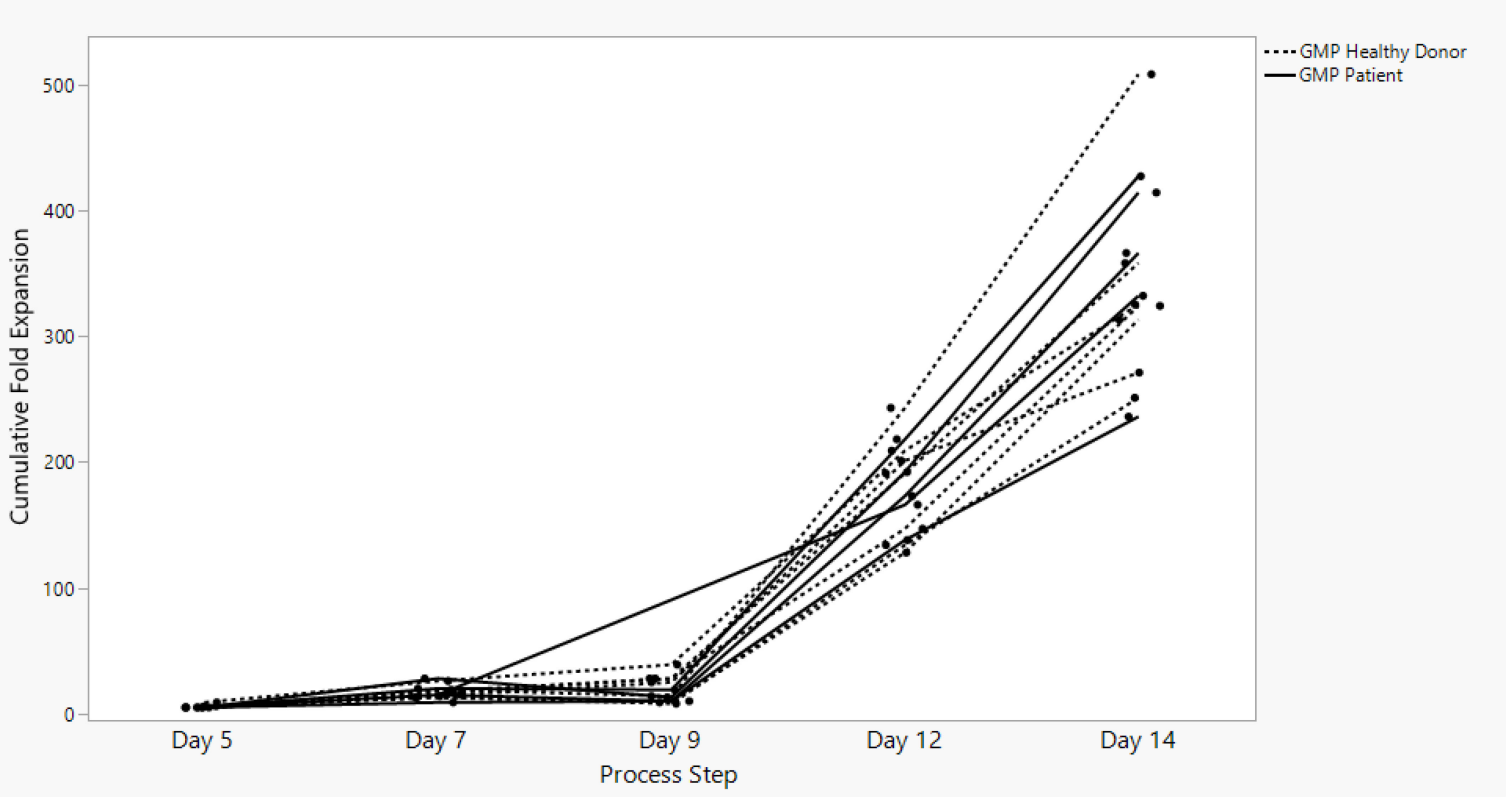
SBT-77–7101 manufacturing achieves significant cumulative fold expansion throughout process duration.

Sonoma Biotherapeutics has developed a manufacturing process for the generation of a chimeric antigen receptor regulatory T-cell product that consists of an ex vivo expanded autologous CD4^+^CD127^lo/-^CD25^+^ T-cell culture that has been transduced with a self-inactivating lentivirus vector encoding a CAR. A partnership with a contract manufacturing organization (CMO) has been established with successful technical transfer and initiation of clinical manufacturing. The process demonstrates high viability and cell health throughout the process (**Figure 1**) and robust cell proliferation, since the number of cells at the start of the process is rate limiting. The current process enables robust cell growth throughout the expansion period, currently 14 days, which is enhanced based on multiple rounds of activation (**Figure 2**). The resulting product exhibits a 250 to 450 cumulative fold expansion (**Figure 3**).

### 2.3 Manufacturing control strategy

Regulatory compliance is critical in the manufacturing of Treg therapies, which fall under the broader category of advanced therapy medicinal products (ATMPs). These include cell-based therapies regulated by the FDA under the Center for Biologics Evaluation and Research (CBER). The manufacturing process for Treg cell therapy must align with current Good Manufacturing Practices (cGMP) and FDA guidelines to ensure safety, potency, purity, and consistency of final product.

There are six core components Sonoma Biotherapeutics follows to ensure our manufacuturing process of Treg cell therapy is in alignment with FDA guidance.

**Donor screening and cell sourcing**

FDA guidelines require comprehensive donor eligibility determination, including infectious disease screening and history assessment (21 CFR Part 1271). Treg therapies often originate from autologous or allogeneic sources, so traceability and donor qualification are key regulatory components.

**Process standardization and control**

Manufacturing processes must be clearly defined and controlled. This includes cell isolation, expansion, and potential genetic modification. Process controls are critical to ensure product consistency. FDA guidance emphasizes the use of well-characterized reagents, closed system processing where possible, and scalable, reproducibly methodologies.

**Product characterization and potency**

Treg products must be thoroughly characterized to demonstrate identity, purity, and potency. This includes phenotypic markers (e.g., CD4+, CD25+, FOXP3+) and functional assays confirming suppressive activity. The FDA requires validated potency assays as a measure of clinical efficacy.

**Safety testing**

In-Process and final product testing for sterility, endotoxins, mycoplasma, and adventitious agents is required. The risk of tumorigenicity or off-target effects is also assessed, especially in gene-edited or expanded Treg populations.

**Documentation and quality systems**

Robust documentation, including batch records and deviations, is essential. A quality management system (QMS) should oversee all aspects of the manufacturing process, from raw material control to final product release, aligning with FDA expectations (21 CFR Parts 210, 211, 600).

**Regulatory submissions and ongoing oversight**

Treg therapies require an Investigational New Drug (IND) application for clinical trials, with comprehensive Chemistry, Manufacturing, and Controls (CMC) documentation. Ongoing dialogue with the FDA through Pre-IND meetings and adherence to regulatory updates is essential for compliance and eventual Biologics License Application (BLA) approval.

The goal of product development is to develop processes that ensure that the drug product being manufactured has the defined product quality characteristics for patient administration (14). A product control strategy ensures the drug product is safe, effective, and consistent between batches (15).

#### 2.3.1 Critical Quality Attributes and critical process performance attributes

CQAs are characteristics of drug products that are essential for their safety, efficacy, and quality, while CPPs are critical parameters that must be controlled during the manufacturing process to ensure the production of a high-quality product (16). Monitoring and controlling these attributes during the manufacturing process is essential to ensure patient safety and therapeutic effectiveness of the drug product. Cross functional drug product development teams, comprised of Process Development, Analytical Development, Product Sciences, Translational and Research work together to determine critical quality attributes (CQAs) and Critical Process Performance attributes (CPPs) of the final drug product. Critical Quality Attributes (CQAs) are measurable properties that define the identity, potency, purity, safety, and stability of the final Treg cell drug product.

#### 2.3.2 Analytical control strategy

Product and process understanding lead to the identification of CQAs requiring analytical measurement for control which are described in a quality target profile (17, 18). The analytical control strategy involves the development and validation of methods to measure CQAs and CPPs. These methods are used to monitor the product’s quality and consistency between batches (19).

The first step in a robust method development workflow is the definition of the analytical target profile (ATP). An ATP consists of a description of the intended purpose, appropriate details on the product attributes to be measured and relevant performance characteristics with associated performance criteria. The ATP includes the performance requirements for a single attribute or a set of quality attributes. The ATP drives the choice of analytical technology and is determined to be suited for their intended purpose by performing method qualifications (19).

Identity markers are particularly critical to ensure the enrichment of a pure and stable Treg cell population. Key Treg surface markers include CD4^+^, CD25^+^, FOXP3^+^, and CD127^low/−^, which collectively help distinguish Tregs from effector T cells. Tregs share several surface markers and soluble factors with activated effector T cells, making potency assays and measures of cellular identity of product difficult. The development of potency assays for Treg cell therapies is a critical step in ensuring product consistency, efficacy, and safety, particularly as these therapies advance in the clinic. Given the multifaceted nature of Tregs and their mechanism of action, primarily immunosuppression, potency assays must capture their functional characteristics while also being feasible for routine use in clinical manufacturing and quality control settings.

Sonoma Biotherapeutics is looking into multiple approaches for understanding Treg Potency: Suppression assays, cytokine production profiles, and FOXP3 TSDR Demethylation. The gold standard for assessing Treg function remains the *in vitro* suppression assay where the Treg cell product is co-cultured with the target population to measure the extent to which Tregs suppress their proliferation or cytokine production in response to activation. Tregs are characterized by low production of pro-inflammatory cytokines (e.g., IFN-γ, IL-2) and may secrete immunomodulatory cytokines like IL-10 and TGF-β. The assessment of epigenetic stability, specifically the demethylation status of the FOXP3 locus at the Treg-Specific Demethylated Region (TSDR), could be keen for determining functional stability of Tregs. Transcriptomic profiling could also aid in identifying unique Treg fingerprint signatures indicative of a pure, stable Treg with immunosuppressive capacity at the gene expression level, characterizing both desired and undesired cells in a final drug product.

There is no universally accepted standard for Treg potency assays, largely due to the complexity and diversity of Treg functions. Multiparametric approaches are often adopted – combining phenotypic markers, functional assays, and molecular signatures. Analysis of characterization of Treg product will drive the understanding of critical attributes for this field. Leveraging these learnings and associated correlations of process levers go hand in hand connecting CQAs and CPPs.

## 3 Treg cell therapy manufacturing: challenges

### 3.1 Manufacturing effectiveness

As described above, the engineered Treg cell therapy manufacturing space is still in early stages, with challenges that remain to be addressed (20). To identify and prioritize focus areas for improving manufacturing effectiveness, Sonoma Biotherapeutics took a risk-based approach. Unit operations were analyzed for areas that would have the following impacts to clinical success:

- Process Performance: measure of how efficient or effective a process is
- Process Control: Active change of the process based on results of process monitoring
- Process Development: improved understanding and ability to manufacture a product in required quantity
- Process Quality: Incorporate features that have capacity to enable product needs
- Manufacturing Success: Production and delivery of quality products
- Innovation: New method, idea, product for continuing success

Scalability of the Treg manufacturing process is a key attribute that would benefit from improvement. Limitations of the current processing dynamics will have an impact on the ability to expand into this novel therapeutic market if unable to navigate the constraints on the ability to adapt to increased demand without sacrificing process performance and efficiency (21, 22). Two key factors contribute to the risk of Treg manufacturing scalability: closing the manufacturing process and enabling high yield of product.

#### 3.1.1 Managing apheresis and developing a stable process

**Figure 4:**
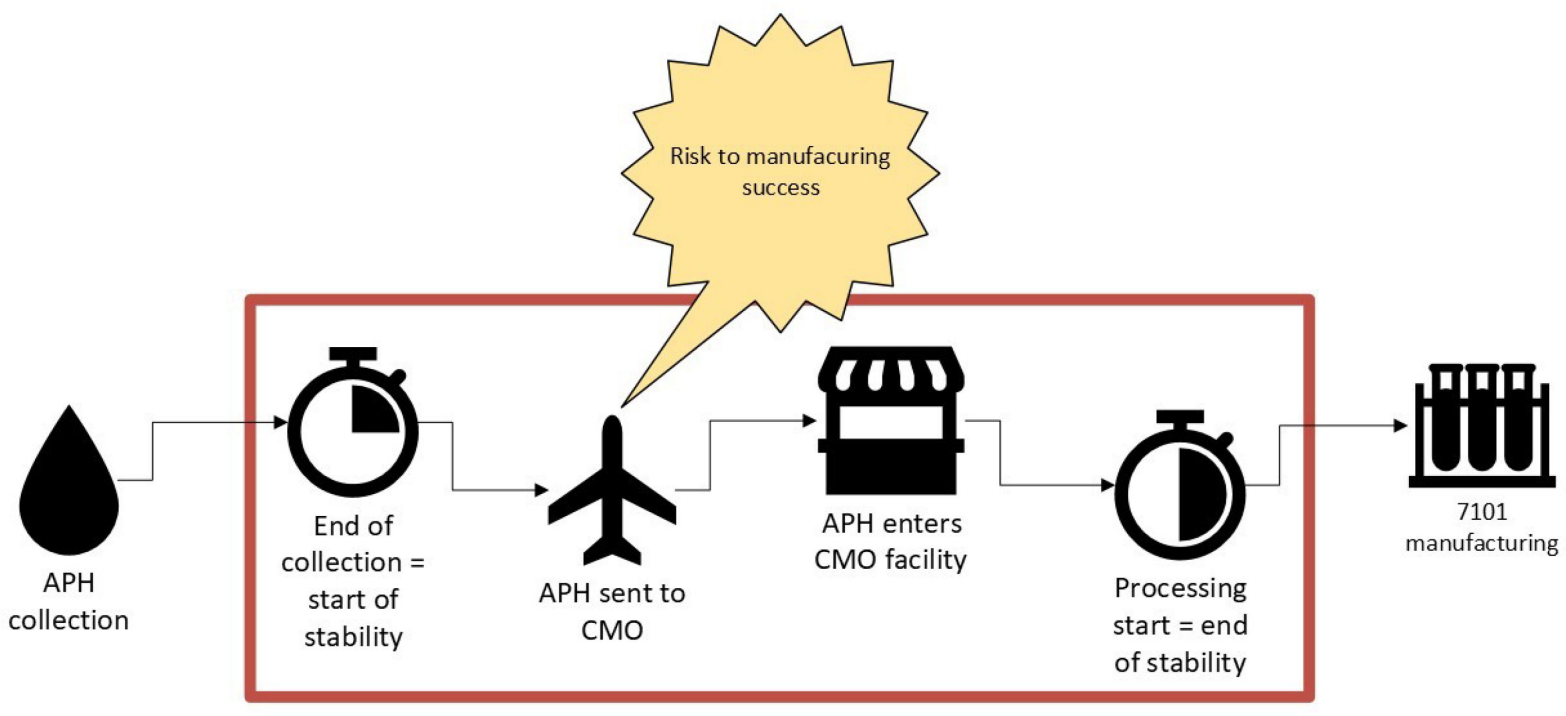
Increasing stability window of starting material will mitigate autologous manufacturing risk; providing confidence and flexibility.

Apheresis is a collection procedure where blood is separated to collect specific blood components (ex: blood cells, white blood cells, platelets and plasma). Leukapheresis is a type of apheresis collection focused on collecting leukocytes from the blood product. The use of freshly collected leukapheresis material is commonly used in the autologous cell therapy field. However, the use of fresh leukapheresis can add substantial constraints to the manufacturing and supply chain workflows as it is critical to coordinate activities across multiple locations and teams in a small window of time (14). Significant coordination and tight monitoring of the starting cellular material is required to ensure efficient delivery from the collection site to the manufacturing facility (**Figure 4**). There is also substantial control on operator scheduling within this narrow window to ensure the manufacturing suite is prepped and processing can begin immediately (2). Importantly, although in the oncology space, apheresis is commonly used and hematology/oncology departments are well organized to conduct the collection procedure, as cell therapy moves into other disciplines, such as rheumatology and dermatology, coordination is more challenging, and increased education is needed to make this aspect of manufacturing more manageable.

Understanding the impact of duration post collection to the start of manufacturing is important to ensure high quality products are obtained with no impact on process performance. To improve and enable flexibility within the manufacturing landscape, extending the acceptable duration of time from end of leukapheresis collection to start of processing is a huge improvement for manufacturing efficiency (4). Extending the stability of starting patient cellular material will reduce the risk of delaying lot release and delaying delivery of the final drug product to patients, ensuring deviation closure or maintaining product quality (23). Furthermore, an extended stability window will allow for increased flexibility in manufacturing and coverage of excursions.

To establish a stability window for starting material, studies should be designed to characterize product quality and process performance throughout the duration you are interested in establishing (24). A common operating window for fresh cellular leukapheresis material is generally within 24 to 48 hours from collection to start of processing (25). Of course, this window heavily depends on the collection protocol, composition of the leukapheresis collection and parameters of your manufacturing workflow. Parameters to consider are to look at shipping conditions of your starting material – ensuring a controlled temperature environment as well as impact of extended hold durations on cell health at the beginning of the manufacturing process (26). Once a tolerated timeframe is narrowed down, studies are executed to observe process performance parameters across an array of donors to ensure that the target APH stability window does not have any negative impact on process as well as product quality (27).

Product quality is equally important to better understand processing hold time of the starting cellular material with the quality of the final drug product. To ensure a thorough understanding of product quality and expansion capability, the following attributes should be assessed: Purity, Transduction efficiency, Strength, Safety (28). It is essential that with any process modifications, high purity of the Treg phenotype of interest is maintained (7).

Autologous manufacturing effectiveness can be improved to a limit with the extension of the APH collection stability window. Extending the starting material stability window can increase the flexibility in manufacturing and supply chain logistics as well as reducing the risk of delaying lot release and delivery of drug product to patients. Future development will be key to continue to address the starting material stability, product quality, and expansion concerns from using fresh leukapheresis in the autoimmune cell therapy space. Establishing different autologous starting patient material sources including whole blood, frozen leukapheresis, or frozen intermediate product (post cell sorting), will enable flexibility in manufacturing across varied cell populations which will be key to the scalability and access of autologous Treg cell therapy.

#### 3.1.2 Evolving the manufacturing process

As teams work to advance cell therapy manufacturing, it is imperative to ensure the design of a process that is phase appropriate and flexible for improvements to ensure manufacturing efficiency. The Engineered Treg Cell Therapy process is cutting-edge methodology requiring a deep understanding of key phenotypes and growth characteristics of these specific T-cell subsets progress (5). At the forefront of early-stage drug product development it is responsible for teams to not over design a manufacturing process. The engineered Treg manufacturing field is at an inflection point in process development learning from clinical manufacturing experience and will now need to start core improvements to advance this therapy to later stages of development. While it is certainly advantageous to have a fully closed, automated and streamlined end-to-end manufacturing system in place as one advances in clinical trial design and march toward a commercial asset, it is important to ensure flexibility and room for core modifications of a process early on as one learns about the specific cell product and the needs of the therapy (29).

Closing the manufacturing process is the first step in this next stage of development (30). Closing the manufacturing process is being approached in several ways. First, is the concept of a fully integrated closed process that will enable an automated effort from vein-to-vein to obviate any open space. Companies such as Cellares, Lonza, and others are striving for this approach. However, for complex processes, such as Treg cell therapies, these systems will take time to put in place and validate. The other approach is to address each unit operation in creating closed components that mitigate contamination events, reduce human error and increase control of the manufacturing workflow (30). The first step in this development plan is to perform a GMP feasibility assessment to determine areas of risk that can be stored to modified in a tiered fashion. The second step in the development workflow is to assess technologies and materials already established in the cell therapy manufacturing field to find solutions to mitigate high scoring areas of risk.

A common hurdle amongst the autologous cell therapy field is the need to expand the desired cell product utilizing common cell culture practices. The strategy employed for this novel Treg field was to utilize core cell culture and cell therapy manufacturing best practices while investing time in innovative development areas to get Treg therapies to clinic (31). Currently teams are in the process of assessing the wide breadth of commercially available closed processing tools for the expansion of Treg cell subsets, automated cell washing, debeading, formulation and fill of final product.

#### 3.1.3 High cell yield

Phase I clinical programs are designed to understand product safety, with dose escalation cohorts. This can be a challenge from a process development perspective as the developer is challenged with delivering a manufacturing process which can enable a high yield of final product that is of high quality (15). This is particularly challenging in the field of Treg cell therapy requiring the need to begin with a highly pure cell phenotype that is not readily abundant in the starting material obtained during patient apheresis collection. As denoted earlier, apheresis material is composed of approximately 1% Treg cell subset (32). During the enrichment and purification step this results in a very low number of starting cells entering the manufacturing process as compared to traditional T_eff_ cell therapies.

There are many factors that confound manufacturing capability on top of the substantially low starting cell number. These components include common characteristics across the autologous cell therapy space as a consequence of uncontrolled donor variability (15). There is a unified challenge across the autologous cell therapy industry of navigating limited control of the incoming starting material. Unlike traditional biologics or even allogenic cell therapy, one must define a manufacturing process that can withstand variability from donor to donor. Two key common donor derived variabilities are transduction efficiency and cell expansion kinetics (23).

The process development team must work alongside manufacturing teams to assess the GMP manufacturing process to identify areas of improved efficiency to maximize cell yield throughout every step of the process (28). Initiatives will include a diverse range of topics spanning areas of cell loss from patient collection through cryopreservation. Key areas and practices in the GMP workflow are identified and optimized to maximize batch records and operational efficiencies. There are benefits in making incremental improvements no matter how small or inconsequential that can lead to profound impacts. An important reminder is that process design and development doesn’t always require a pivot to new innovative technology to improve efficiency.

## 4 Treg cell therapy manufacturing: new and future opportunities

### 4.1 Process & analytical improvements: platform based & integrated automation

The development of engineered Treg therapies is complex and multifaceted, necessitating continuous enhancements in process efficiency and analytical capabilities. To meet the growing demands for scalability, consistency, and regulatory compliance, organizations are increasingly adopting platform approaches and leveraging automation in their processes.

#### 4.1.1 Platform approach

The platform approach entails the development of standardized processes that can be applied across multiple programs or products. By establishing a unified platform for engineered Treg cell therapy production, organizations can streamline workflows, reduce variability, and enhance reproducibility (33). This standardization facilitates quicker technology transfer, as teams can leverage existing protocols and workflows without the need for extensive retraining or systems overhauls.

Platforms are designed to be modular and adaptable, allowing organizations to scale production based on demand while maintaining consistency in quality (34). A platform-based framework can accommodate various cell types or genetic modifications, thus minimizing the time and resources necessary for process development (33). Such flexibility is essential in the rapidly evolving landscape of cell and gene therapies, where the ability to pivot between different product candidates can enhance market competitiveness.

By integrating analytical methods into a standardized platform, organizations can enhance their testing and characterization processes. This integration enables simultaneous assessment of process parameters and product quality attributes, ensuring compliance with regulatory standards (26). Utilizing a platform approach also facilitates the early identification of potential issues during the manufacturing process, allowing for timely interventions and process optimization.

#### 4.1.2 Automation

Automation technologies, including robotic systems and automated liquid handling platforms, would significantly enhance operational efficiency in the engineered Treg cell therapy production. Automation streamlines repetitive tasks, such as cell culture handling, expansion, and harvesting, leading to reduced manual intervention and improved throughput (2). This increase in operational efficiency not only accelerates production timelines but also minimizes the risk of human error, thereby enhancing the overall quality of the final product.

The use of automation systems facilitates real-time data acquisition and analysis, allowing organizations to monitor process parameters continuously (35). This capability aids in identifying deviations or trends early in the production cycle, enabling data-driven decision making and rapid corrective actions. Advanced data integration platforms can create a centralized repository for process and product data, enhancing traceability and compliance while also supporting ongoing process improvements through analytics (15).

Incorporating automation enables the implementation of sophisticated process control strategies, including feedback loops and predictive analytics (23). These systems can dynamically adjust process parameters based on real-time data, ensuring optimal conditions for cell growth and product consistency). Such advanced control systems can also lead to significant reductions in variability, ultimately contributing to higher yields and more reliable product quality.

The adoption of a platform approach and the integration of automation technologies represent significant advancements in the development of engineered Treg cell therapies. By standardizing processes, enhancing scalability, and leveraging automation for increased efficiency and improved data management, organizations can not only streamline their manufacturing operations but also enhance product quality and compliance (8). As the field of cell therapy continues to evolve, embracing process and analytical improvements will be crucial to meeting the demands of the market and ensuring the successful delivery of innovative therapies to patients.

### 4.2 Contract manufacturing organization and contract research organization management

In the development of engineered Treg cell therapies, effective management of Contract Manufacturing Organizations (CMOs) and Contract Research Organizations (CROs) is paramount. Organizations need to make an early choice between building internal manufacturing capability and testing facilities or working with contract organizations. Small biotech operations often opt for partnerships to preserve capital and build cross-functional expertise. In cell therapy, the process is the product. Finding an ideal CMO/CRO partnership is crucial. These partnerships can significantly influence the success of technology transfer, process development, compliance and overall project timelines. A systematic approach to vendor management encompasses establishing strong vendor relationships, ensuring effective technology transfer, facilitating comprehensive training and maintaining open communication channels (36).

Vendor relationship management should be established and actively monitored. Establishing a solid foundation of trust and collaboration with CMOs and CROs is essential for fostering successful partnerships (37). Organizations should prioritize aligning goals, expectations and timelines with their vendors from the outset. Regular face-to-face meetings, consultations and joint planning sessions can help nurture these relationships, encouraging proactive problem solving and collaborative innovation. Performance metrics should be established and monitored. Defining key performance indicators (KPIs) to evaluate vendor performance is vital. Metrics may include timeline adherence, production runs, product quality and compliance with regulatory requirements (36). Regular performance reviews can help identify areas for improvement and ensure that both parties remain accountable. Feedback loops should be established to maintain transparency and continuous improvement.

Technology Transfer is a key foundation of success that should be structured and well planned. Implementing comprehensive technology transfer plans is essential to facilitate effective knowledge transfer between the organization and its vendors. These plans should detail the specifics of the processes, methodologies and equipment involved in the Treg engineered Treg cell therapy production and release testing (38). Documentation should include batch records, standard operation procedures (SOPs) and validation protocols to ensure that all parties have the requisite information to replicate processes accurately (36). Engaging cross-functional teams that include members from R&D, manufacturing, quality assurance and regulatory affairs fosters a holistic approach to technology transfer. This collaboration ensures that each aspect of the process is addressed, and that expertise is shared seamlessly. Regular meetings between internal teams and external vendors can help to synchronize efforts, manufacturing and testing challenges, and align expectations.

Comprehensive training programs and ongoing education should be a priority focus to ensure success. A robust training program tailored for both internal stakeholders and vendor personnel is crucial for successful technology transfer (38). This training should encompass the specifics of engineered Treg cell therapy manufacturing, quality control measures and regulatory compliance. Training sessions can include hands-on workshops, online modules and demonstrations to ensure that all personnel have a thorough understanding of the processes involved (37). Continuous education efforts are essential for keeping vendors updated on advancements in technology and regulatory changes. Providing ongoing support through refresher courses and knowledge sharing platforms fosters a culture of growth and learning. Encouraging vendor teams to participate in relevant conferences and workshops also enriches their expertise and strengthens the partnership.

Establishing open lines of communication is critical for fostering collaboration and transparency throughout the vendor relationship. Regular updates and check-ins with both CMOs and CROs allow for timely identification and resolution of potential issues. Utilizing digital communication platforms can enhance information sharing, streamline workflows and facilitate rapid decision-making.

In summary, effective CMO/CRO vendor management is an essential component of successful engineered Treg cell therapy clinical development. By fostering strong vendor relationships, establishing structured technology transfer plans and implementing comprehensive training programs with open communication can streamline workflows to effectively manage costs.

### 4.3 Supply challenges

The development and commercialization of engineered T cell therapies come with various supply chain challenges, particularly regarding sourcing materials and components essential for manufacturing. Among these challenges, reliance on single or sole-source suppliers presents significant risks that can affect overall project timelines, product availability, and compliance with quality standards (39).

Implications of a single or sole source are substantial and numerous spanning supply disruptions, pricing and regulatory compliance. Relying on a single supplier for critical materials, such as vector systems, growth factors, or starting materials (e.g., peripheral blood mononuclear cells), increase vulnerability to supply disruptions (40). Any interruption in this supply due to manufacturing issues, quality control failures or logistical challenges can halt production and delay clinical trials. Single sourcing can also limit competitive pricing dynamics, leading to potentially higher costs for essential inputs (39). Without competition, suppliers may have less incentive to keep prices in check, impacting overall production costs. Regulatory authorities often emphasize the need for a robust supply chain with verified backups. Sole-sourced arrangements may raise concerns during the regulatory review process as regulators may require comprehensive justification for the reliance on a single entity (39).

Strategies to mitigate these supply challenges involve the diversification of suppliers and risk assessment and contingency planning (41). Establishing relationships with multiple suppliers can help mitigate the risks associated with sole source dependencies where available. By creating a diversified supply chain, organizations can better ensure continuity of materials and components, thereby reducing the likelihood of disruptions. Strategic partnerships with alternative suppliers can also lead to increased bargaining power and favorable pricing arrangements. Developing a comprehensive risk assessment framework can help organizations identify critical supplies and evaluate their reliance on single or sole-source suppliers (41). Defining contingency plans that outline steps to take in the event of supply disruptions, ensuring that alternative sourcing strategies are ready to be implemented.

In the context of engineered Treg therapy development, supply challenges such as reliance on single or sole-source suppliers must be navigated carefully. By diversifying supply sources, establishing robust contracts, conducting thorough risk assessments and maintaining collaborative relationships, organizations can better manage these challenges and successfully deliver live-saving therapies to patients.

## 5 Discussion

Above is a comprehensive overview of the current state of the engineered Treg cell therapy field, focusing on essential manufacturing challenges. With the continuing rise in interest in personalized medicine within the immuno-oncology sector, understanding and optimizing the manufacturability of Treg CAR-T therapies is critical for successful clinical outcomes and market viability. One of the primary themes discussed is scalability. The manufacturing process for engineered Treg cells is still in its infancy, characterized by labor-intensive steps and reliance on specialized equipment. The demand for higher patient throughput necessitates the development of automation and integrated unit operations, which could streamline production and reduce waste. While current approaches can yield viable cell populations from diverse patient samples, optimizing the scalability of these processes without compromising quality remains a challenge.

The need for effective cell sorting and isolation is vital for maintaining the purity of Treg populations. Existing technologies currently fall short of effectively isolating these rare cells. As Treg subsets are identified by multiple markers, advancements in multiparametric selection techniques, particularly flow cytometry, are essential. Continuous investment in operator training to proficiently utilize these complex systems is equally crucial to avoid complications in the cell therapy timeline.

Stability is another central component of manufacturability. The ability to extend the stability window for apheresis material is a significant breakthrough, providing operational flexibility. By coordinating logistics and ensuring optimal conditions for cell health, manufacturers can reduce the risk of delays caused by lost stability during transport. This aspect highlights the importance of a robust analytical strategy that correlates processing parameters to product quality.

The implementation of comprehensive product control strategies encompassing critical quality attributes is key. By integrating these practices early in the clinical trial process, manufacturers can refine their analytical methods to ensure consistent product safety and efficacy. The emphasis on characterizing the product during various development phases indicates a growing recognition of the role that analytics play in maximizing product integrity.

Cost considerations loom large in the development of novel therapies. Here we address the inherent expenses related to engineered Treg cell therapy and suggest cost-effectiveness through the optimization of processes can yield substantial benefits, especially in improving patient accessibility to these cutting-edge therapies. Establishing partnerships with CMOs and CROs is critical for balancing the cost-benefit ratio in the commercial sector. The systematic approach to vendor management proposed here aligns well with current trends in the biopharma sector, stressing the need for agility and collaboration.

Looking ahead, the potential for a platform-based approach combined with automation could revolution engineered Treg therapies. Standardizing process can mitigate variability and enhance reproducibility thereby facilitating regulatory compliance. The integration of data analytics tools alongside automation can drive continuous improvements thereby accelerating the pace of innovation in this field.

In conclusion, we present a proactive roadmap for addressing current challenges in the autologous engineered Treg cell therapy manufacturing space. By focusing on scalability, stability and cost-efficiency we have laid out the groundwork for future advancements that could significantly enhance the accessibility and effectiveness of these live altering therapies. As research progresses and technologies evolve, the field can expect not only improved clinical outcomes for patients but also a more streamlined manufacturing pipeline that aligns with growing demands of the healthcare market.
